# Impact of Nanoplastic Particles on Macrophage Inflammation and Intestinal Health in a Mouse Model of Inflammatory Bowel Disease

**DOI:** 10.3390/nano14161350

**Published:** 2024-08-15

**Authors:** Marlene Schwarzfischer, Tano S. Ruoss, Anna Niechcial, Sung Sik Lee, Marcin Wawrzyniak, Andrea Laimbacher, Kirstin Atrott, Roberto Manzini, Marijn Wilmink, Luise Linzmeier, Yasser Morsy, Silvia Lang, Gerhard Rogler, Ralf Kaegi, Michael Scharl, Marianne R. Spalinger

**Affiliations:** 1 Department of Gastroenterology and Hepatology, University Hospital Zurich, University of Zurich, 8091 Zurich, Switzerland; marlene.schwarzfischer@usz.ch (M.S.); marianne.spalinger@usz.ch (M.R.S.); 2 Scientific Center for Optical and Electron Microscopy, ETH Zurich, 8093 Zurich, Switzerland; 3 Institute of Biochemistry, ETH Zurich, 8093 Zurich, Switzerland; 4 Department Process Engineering, Swiss Federal Institute of Aquatic Science and Technology (Eawag), 8600 Dübendorf, Switzerland

**Keywords:** nanoplastic, intestinal homeostasis, macrophages, inflammatory bowel disease

## Abstract

Background: The increasing presence of plastics in the human diet is raising public concern about the potential risks posed by nanoplastic (NP) particles, which can emerge from the degradation of plastic debris. NP ingestion poses particular risks to individuals with inflammatory bowel disease (IBD), as compromised epithelial barriers may facilitate NP translocation. Methods: In vitro, bone-marrow-derived macrophages (BMDMs) were exposed to 25 nm polymethacrylate (PMMA) or 50 nm polystyrene (PS) particles to assess morphological changes and alterations in pro- and anti-inflammatory gene expression. In vivo, mice received PMMA NP particles for 6 months before acute dextran sodium sulfate (DSS) colitis was induced to investigate NP impacts on intestinal health and inflammation. Results: PMMA and PS NP exposure in BMDMs induced morphological changes indicative of a proinflammatory phenotype characterized by enlarged amoeboid cell shapes. It also triggered an inflammatory response, indicated by increased expression of proinflammatory cytokines such as *Tnfa* and *Il6*. Unexpectedly, long-term PMMA NP administration did not affect the intestinal epithelial barrier or exacerbate acute DSS-induced colitis in mice. Colonoscopy and histological analysis revealed no NP-related changes, suggesting adverse effects on intestinal health or inflammation. Conclusion: Our findings from animal models offer some reassurance to IBD patients regarding the effects of NP ingestion. However, variations in lifestyle and dietary habits may lead to significantly higher plastic intake in certain individuals, raising concerns about potential long-term gastrointestinal effects of lifelong plastic consumption.

## 1. Introduction

After decades of environmental pollution with various plastic litter, concerns are growing that the rising global plastic burden might affect marine and terrestrial life. However, production and consumption of plastics are continuously increasing [1], leading to the accumulation of plastic debris around the globe [2]. Particular attention is being given to small plastic particles which can derive from different sources; primary plastic particles are introduced into freshwater and saltwater as manufactured microscale polymers, commonly found in cosmetic care products and medications [3], while secondary particles are generated through the ongoing fragmentation of plastic waste [4]. A significant portion of secondary plastic particles originates from public litter, improper waste disposal by the fishing industry [4], sewage water containing plastic particles from washing synthetic textiles [5], and tire abrasion [6]. Environmental impacts lead to the creation of microplastic (MP) particles measuring less than 5 mm in diameter, which can subsequently break down into even smaller nanoplastic (NP) particles [7]. The definition and size range of nanoplastics remain subjects of debate within the scientific community. While the commonly accepted size limit is under 100 nm, some extend this range up to 1000 nm. Plastic residues have been found extensively worldwide, spanning beaches, shores, freshwater and saltwater bodies, deep-sea sediments, and even within Antarctic sea ice [7].

Although most studies examining the impact of plastic particles on the environment have focused on the marine ecosystem, plastic contamination may be more widespread in the terrestrial ecosystem and human diet than previously anticipated. Small plastic particles are consumed by aquatic organisms [8], entering the circulatory systems of marine wildlife. Subsequently, they propagate throughout the marine food chain, ultimately contaminating seafood intended for human consumption [9]. Moreover, plastic packaging used for beverages and food is an obvious source of ingested NP and MP particles [10]. Thermal stress can induce the migration of small plastic particles into food; however, exposure estimates need to be validated and testing methods need to be standardized in order to perform comprehensive risk assessments [11]. However, MP particles have also been detected in tap water, honey, sugar, sea salt, and beer [12,13,14,15,16]. It is estimated that an average adult consumes approximately 39,000 to 52,000 particles per year [17]. Other studies estimate that humans consume between 0.1 and 5 g of plastic each week, which is roughly equivalent to a credit card-sized amount of plastic [18]. Unsurprisingly, microplastics have been detected in human feces [19] and blood [20]. 

NP particles possess high bioavailability due to their small diameter [21,22]. It is expected that synthetic particles with a diameter below 130 µm can cross the gastrointestinal barrier via paracellular transport [21,23], while particles smaller than 10 µm may be absorbed by microfold cells [21,24], where they are passed to intestinal macrophages. Macrophages are immune phagocytes with the capacity to efficiently clear foreign-body particles. In the gastrointestinal tract, macrophages patrol along the intestinal epithelium, where they sense and eliminate bacterial products and foreign-body particles that surpass the intestinal epithelial barrier [25]. They play crucial roles in the innate immune system and maintain immune balance. Thus, macrophages are at the front line of host defense and are expected to be the first immune cells to encounter ingested NP or MP particles in the intestine. However, the impact of NPs and MPs on macrophages still needs to be investigated.

Despite the rising global abundance of small plastic particles and increasing awareness, plastic research is still in its early stages, and the potential impact of exposure to NPs and MPs on human health remains unpredictable. As the consumption of small plastic particles continues to increase along with rising levels of plastic in the human diet, the toxicity risks could escalate due to the accumulation of these particles in the body. Since particles with smaller diameters are believed to penetrate epithelial barriers more easily [21,24]; nanosized plastic particles are anticipated to have high bioavailability, facilitating increased translocation into the system.

Thus, studying the effects of plastic exposure on human health is of great public interest. Individuals with chronic intestinal inflammation, such as inflammatory bowel disease (IBD), could be especially susceptible to the harmful effects of ingested plastic particles. This vulnerability arises because disruptions and increased permeability of the intestinal epithelial barrier [26] may enable greater translocation of MP and NP particles into the systemic circulation compared to healthy individuals. While initial in vivo studies report proinflammatory and adverse health effects in rodents upon oral exposure to MP particles [27,28,29,30,31], data on NP particles are very limited and require closer investigation. Here, we evaluate the impact of NP particles on macrophages and intestinal health in homeostasis and during the development of intestinal inflammation.

## 2. Materials and Methods

### 2.1. Ethical Considerations

Animal experiments were carried out in compliance with Swiss animal welfare laws and approved by the local veterinary office (Veterinary Office of the Canton Zurich; License 019/2018). Wildtype mice on a C57BL/6 background were bred and housed in a specific pathogen-free (SPF) facility with unrestricted access to standard chow and water.

### 2.2. Isolation and Cultivation of Bone Marrow-Derived Macrophages

Bone marrow was isolated from 8-week-old mice, homogenized with an 18 gauge needle, and passed through a 70 µm cell strainer, as previously described [32]. Cells were seeded in noncoated Petri dishes at a density of 3 × 10^6^ cells in 10 mL RPMI 1640 media (Thermo Fisher Scientific, Waltham, MA, USA), supplemented with 10% FCS (#S181T-500, Biowest, Nuaillé, France), 1 mM Sodium Pyruvate (Thermo Fisher Scientific), 1 mM L-Glutamine (Thermo Fisher Scientific), 50 units/mL penicillin and 50 µm/mL streptomycin (Thermo Fisher Scientific), and 20 ng/mL recombinant mouse MCSF (#576408, BioLegend), and cultivated at 37 °C, 10% CO_2_. Media were replaced on day 5 post isolation. On day 7, cells were detached from the Petri dishes using Accutase (StemCell, Köln, Germany) and seeded in 6-well plates at a density of 3 × 10^6^ cells in 5 mL RPMI media per well. Macrophage differentiation was confirmed by flow analysis, quantifying the expression of the pan-macrophage marker F4/80, which was present in 93% of all cells. Bone-marrow-derived macrophages (BMDMs) were exposed to green-fluorescent NP particles (Micromer greenF plain 25 nm polymethacrylate particles #29-00-251, spheric, suspension in water, Micromer greenF plain 50 nm polystyrene particles #29-00-501, spheric, suspension in water, Micromod, Rostock, Germany) diluted in RPMI media at a concentration of 100 µg/mL, 200 µg/mL, and 400 µg/mL. After incubation for 2 h, 4 h, 8 h, or 24 h, cells were rinsed with PBS twice and scraped off the surface in 200 µL homogenization buffer containing thiogylcerol (#AS1390, Promega, Madison, WI, USA). Bright-field images of the treated cells were taken using a Nikon ECLIPSE Ts2 microscope (Nikon, Düsseldorf, Germany).

### 2.3. RNA Isolation, RT-PCR, and Real-Time PCR

RNA was extracted using the Maxwell RSC simplyRNA Tissue kit and Maxwell RSC instrument (Promega, Walldorf, Germany), following the manufacturer’s guidelines. RNA concentration was determined by measuring absorbance at 260 nm and 280 nm using a Nanodrop 1000 spectrophotometer (Thermo Fisher Scientific). Complementary DNA (cDNA) was synthesized using the High-Capacity cDNA Reverse Transcription Kit (Thermo Fisher Scientific). Gene expression analysis utilized predesigned TaqMan Real-Time PCR Assays and TaqManTM FAST Universal PCR Master Mix on a QuantStudio 6 Flex Real-Time PCR System, following the manufacturer’s protocols (all reagents purchased from Thermo Fisher Scientific). Measurements were conducted in triplicate, and results were normalized to murine *Actb* (Thermo Fisher Scientific) as an endogenous control, analyzed using the ∆∆CT method.

### 2.4. IF Staining and Confocal Microscopy

BMDMs were differentiated as described above and seeded on glass coverslips before treatment with green-fluorescent 25 nm or 50 nm NP particles (Micromer greenF plain 25 nm polymethacrylate particles #29-00-251, Micromer greenF plain 50 nm polystyrene particles #29-00-501, Micromod) diluted in RPMI at a concentration of 100 ug/mL. Following a 24 h incubation period, cells were fixed with 4% paraformaldehyde for 20 min, washed three times with PBS, and then blocked in PBS containing 10% normal goat serum (NGS) and 5% bovine serum albumin (BSA). Cells were incubated with Phalloidin iFluor 647 (#ab176759, Abcam, Cambridge, UK) and DAPI diluted in PBS, 1% NGS, and 1% BSA for 1 h at room temperature. Coverslips were washed in PBS three times and shortly dipped in ultrapure water before mounting on glass slides using fluorescence mounting media (Agilent Technologies, Santa Clara, CA, USA). Fluorescent-labeled cells were examined using the LEICA SP8 Upright equipped with a PL Fluotar 40× oil objective (Leica Microsystems, Wetzlar, Germany). Images were processed using the LAS X (Leica) and Imaris 9.3 software (Oxford Instruments, Abingdon, UK).

### 2.5. Mice and In Vivo Treatments

Study design: Appropriate control groups were included in all experiment. Since the DSS and PMMA NP particles were supplied in the drinking water, each cage was considered as an experimental unit. Female C57Bl6 WT mice (3 weeks old) were used for all experiments. Long-term administration: In this setup, a relative low variance was expected within each group. Assuming a difference of 30% between treatment groups and controls, considering 6 groups to be compared, and a required power of 80% including an alpha error of 0.05, ANOVA resulted in a group size of 10 animals per group. Acute DSS colitis: Within this setup, a medium variance was expected within each group. Assuming a difference of 30% between treatment groups and controls, considering 6 groups to be compared, and a required test power of 80% including an alpha error of 0.05, ANOVA resulted in a group size of 20 animals per group. Terminal evaluation of inflammation was performed in a blinded manner. The here-described experiments are confirmatory. Primary outcome: MEICS score (0–15 with 0.5 increments). Additional outcome: Weight change, histology score, colon length, spleen weight, barrier integrity. The null hypothesis posits that there is no difference among the means of all groups. The alternative hypothesis is that at least one of the paired comparisons would change the colitis score. Since colitis induction is influenced by the weight, the mice underwent stratified randomization according to their body weight prior to the start of the DSS treatment. The concentration of DSS used in this study was determined based on our group’s experience and recent experiments. It was selected to induce moderate colitis, enabling the detection of any potential exacerbating effects of PMMA particle administration on the disease.

Termination criteria during the study: Appearance: elevated (orifices clearly clotted, abnormal body posture); behavior: elevated (almost no movement); stool consistency: liquid; blood in stool: rectal blood loss; weight loss since start of the experiment: ≥20%. The sample size was determined by the number of female animals that were retrieved in our breedings. Due to animal welfare reasons we decided to refrain from using additional animals, as plastic administration clearly showed no effect in the conducted experiments.

Sample size: long-term administration: 16 weeks: Ctrl: n = 8, NP: n = 8; 20 weeks: Ctrl = 8, NP = 8; 24 weeks: Ctrl = 8; NP = 9. One mouse from 20 weeks NP and 24 weeks Ctrl had to be removed from the experiment due to elephantiasis. No MEICS score and barrier integrity assay from one mouse 24 weeks NP due to sudden death during anesthesia induction. Acute DSS colitis: H2O: n = 7; DSS: n = 16; NP: n = 8; DSS NP: n = 16. DSS: No MEICS score from one mouse as it died during anesthesia induction. From one mouse in the DSS NP group, a histology score could not be assigned as tissue section could not be evaluated due to technical issues.

Immediately after weaning, mice were supplemented with 25 nm polymethacrylate (PMMA) particles (Micromer plain 25 nm polymethacrylate particles #01-00251, spherical, suspended in water, Micromod). Transmission electron microscopy (TEM) images of the particles are shown in Figure S1A. Nanoparticles (NPs) were provided in the drinking water at a concentration of 0.05 mg/mL, resulting in an average daily consumption of approximately 0.2 mg per mouse, based on an average daily water intake of 4 mL per mouse. For long-term administration, mice were exposed to NP particles for 4, 5, and 6 months with staggered termination. In colitis experiments, mice were pretreated with NP particles for 6 months before induction of colitis. Colitis was induced by administering 1.5% DSS (#9011-18-1, MP Biomedicals, Santa Ana, CA, USA) in drinking water for 7 days, followed by 3 days of normal drinking water. Control mice received normal drinking water with or without PMMA particles but did not receive DSS. NP particles were administered throughout the entire experiment. Female WT mice (n = 8 per condition) were used for in vivo experiments. Average water consumption within each group was monitored daily during the initial 14 days of the experiment and subsequently twice a week. Average consumption of drinking water throughout the experiments is depicted in Supplementary Figure S1C,D. Water bottles of the respective experimental groups were weighed and divided by the number of days between measurements and the number of animals per cage. Prior to DSS administration, control and NP groups were randomized based on body weight. The concentration of DSS used in this study was determined based on our group’s experience and recent experiments, chosen to induce moderate colitis, facilitating the detection of potential exacerbating effects of PMMA particle administration on the disease. Water consumption was not affected by the presence of NP particles or DSS. Drinking water and bottles with or without DSS and NPs were replaced twice a week. Bottles were agitated daily to ensure even distribution of NPs in the drinking water. No biosurfactant was utilized and, according to the manufacturer’s product information, the purchased particles were free of surfactants. NP particles remained well dispersed in the drinking water, as confirmed by dynamic light scattering (DLS) experiments conducted over 48 h (Figure S1B). Since DSL and TEM delivered divergent data with respect to particle size, corona formation around the particles in the drinking water cannot be excluded. However, this cannot be evaluated as the extent of protein/inorganic content in the drinking water of the animal husbandry was not analyzed. Visually, no aggregation or sedimentation of particles was observed during the experiments.

### 2.6. FITC-Dextran Assay

On the final day of the experiment, mice underwent a 2 h fasting period (no food but water ad libitum) before receiving an oral gavage of 200 µL FITC-dextran (80 mg/mL, #46944-500MG-F Fluorescein isothiocyanate-dextran average molecular weight 4000, Sigma Aldrich, St. Louis, MO, USA). Five hours after gavage, mice were anesthetized by intraperitoneal injection of ketamine (90–120 mg/kg body weight, Vétoquinol, Bern, Switzerland) and xylazine (8 mg/kg body weight, Bayer AG, Leverkusen, Germany), and blood was collected retrobulbarly into serum collection tubes. The blood was centrifuged at room temperature for 5 min at 8000× *g* and diluted 1:5 with PBS (pH 7.4). Serum FITC-dextran concentration was measured in triplicate at an excitation wavelength of 485 nm and an emission wavelength of 535 nm. Standard curves for calculating FITC-dextran concentration in the samples were generated by diluting FITC-dextran in serum from nongavaged mice (0, 125, 250, 500, 1000, 2000, 4000, 8000 ng/mL) diluted in PBS.

### 2.7. Assessment of Colitis Severity

Colonoscopy was conducted on anesthetized mice using the Video-Uretero-Renoskop FLEX-X C IMAGE1 STM camera system (Karl Storz SE & Co. KG, Tuttlingen, Germany), following established procedures [33]. Colitis severity was evaluated using the murine endoscopic index of colitis severity (MEICS) scoring system, which considers five parameters: (i) transparency of the colon wall, (ii) changes in the vascular pattern, (iii) presence of fibrin, (iv) granularity of the mucosal surface, and (v) stool consistency. Mice were euthanized to assess spleen weight and measure colon length. Epithelial damage and inflammatory infiltrates were evaluated using H&E-stained sections of the most distal 1 cm of the colon, and scoring was performed according to established methods [34]. Both colonoscopy and histological evaluations were conducted by two independent researchers.

### 2.8. H&E Staining

The distal 1 cm segment of the colon was fixed in 4% formalin and subsequently embedded in paraffin. Sections of 5 µm thickness were deparaffinized using Histoclear (Chemie Brungschwig, Basel, Switzerland) and rehydrated through a descending series of alcohol. H&E staining was conducted following standard protocols and analyzed with Imager.Z2 and ZenPro 2.0 software (Zeiss, Oberkochen, Germany).

### 2.9. Statistics

Statistical analyses were conducted using two- or one-way ANOVA with Tukey’s multiple comparisons for parametric data and the Kruskal–Wallis test for nonparametric data. Results are presented as mean ± SEM, and significance was defined as *p* < 0.05.

## 3. Results

### 3.1. NPs Are Engulfed and Cause Phenotypic Changes in BMDMs

**Figure 1:**
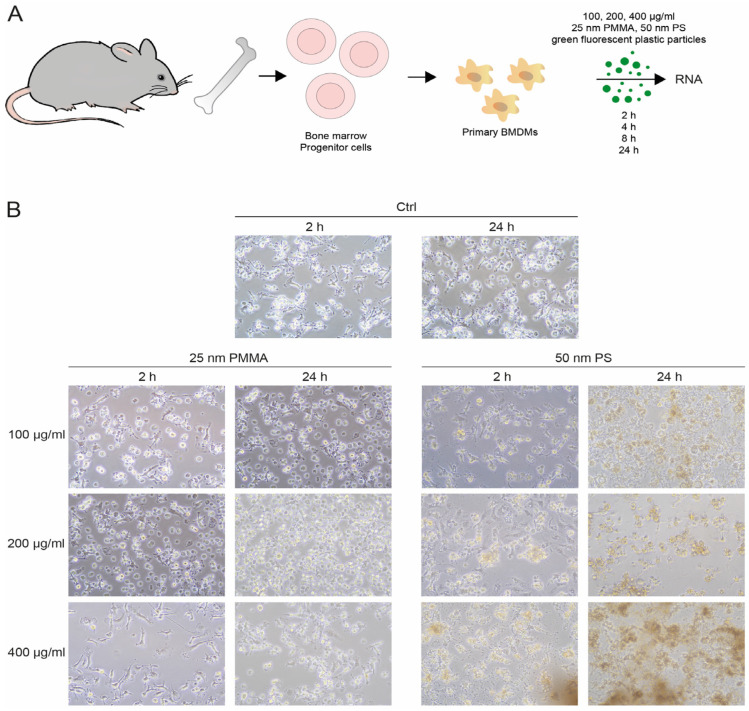
NP particles caused phenotypic changes in BMDMs. Bone marrow was isolated from 10-week-old WT mice and cells were differentiated into BMDMs before treatment with 25 nm PMMA or 50 nm PS particles at a concentration of 100, 200, or 400 µg/mL for 2, 4, 8, or 24 h. (**A**) Schematic overview of experimental procedure. (**B**) Bright-field images of BMDMs after NP exposure. Original magnification (BF) 40×.

To examine the impact of plastic exposure on these phagocytes, bone-marrow-derived macrophages (BMDMs) were cultured with fluorescent 25 nm PMMA or 50 nm PS particles for 24 h prior to evaluating alterations in morphology and RNA expression (Figure 1A).

**Figure 2:**
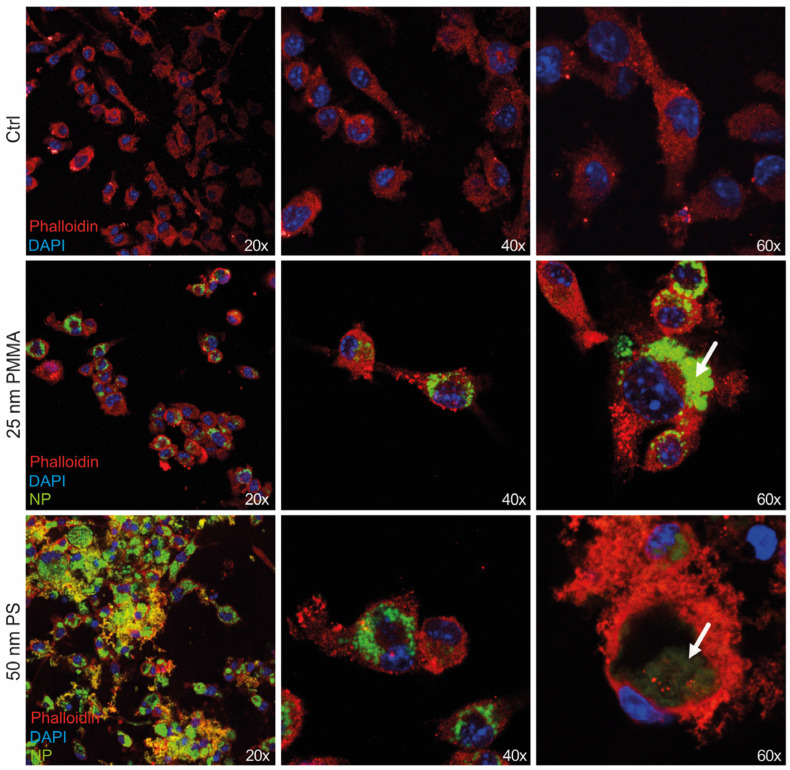
NP particles were engulfed by BMDMs and resulted in rearrangement of the cytoskeleton and phenotypical changes. Bone marrow was isolated from 10-week-old WT mice and cells were differentiated into BMDMs before treatment with green-fluorescent 25 nm PMMA or 50 nm PS particles at a concentration of 100 µg/mL for 24 h. Confocal microscopy of NP (green)-treated cells stained with DAPI (blue) and Phalloidin (red). White arrows indicate enlarged vacuoles.

Bright-field microscopy revealed drastic alterations in macrophage morphology after exposure to NP particles (Figure 1B). In particular, after an incubation time of 2 h, BMDMs treated with NP particles started to detach from the plate and thus became spheric. This effect was even more pronounced with increasing incubation time and more distinct with bigger particle size (Figure 1B). Confocal microscopy revealed that both 25 and 50 nm NP particles were engulfed by the BMDMs, independently of the particle concentration. Also in confocal microscopy, the phenotypical changes in BMDM upon absorption of the NP particles were evident: the particles accumulated in the cytosol, and, especially with 50 nm particles, the macrophages exhibited drastically enlarged vacuoles (Figure 2). This demonstrates that plastic particles were taken up by macrophages and drastically changed their morphology.

### 3.2. NPs Promote Activation and Proinflammatory Response of BMDMs

**Figure 3:**
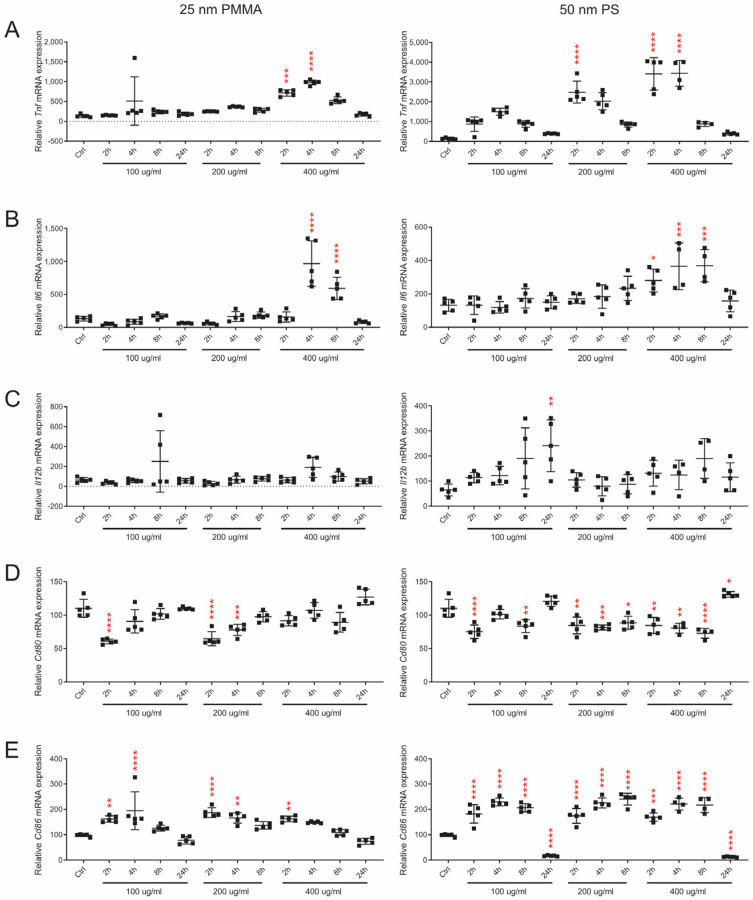
NP administration activated BMDMs and induced proinflammatory cytokine expression. Bone marrow from 10-week-old WT mice was differentiated into BMDMs, then treated with 25 nm PMMA or 50 nm PS particles (100, 200, or 400 µg/mL) for 2, 4, 8, or 24 h. Relative mRNA levels of cytokines (**A**) *Tnfα* (**B**) *Il6*, (**C**) *Il12b*) and costimulatory molecules (**D**) *Cd80*, (**E**) *Cd86*) following exposure to 25 nm (**left**) and 50 nm (**right**) particles are shown. Red asterisks indicate significant differences compared to controls. ∆∆CT method and one-way ANOVA (* = *p* ≤ 0.05, ** = *p* ≤ 0.01, *** = *p* ≤ 0.001, **** = *p* ≤ 0.0001).

NP administration did not only induce morphological changes in BMDMs but also altered cytokine expression levels. In particular, 400 µg/mL NP particles induced expression of *Tnfa* in a time-dependent manner with a significant induction after 2 and 4 h and normalization after 8 h (Figure 3).

Similar effects were observed with *Il6*; however, elevated expression was observed after 4 and 8 h, and levels normalized 24 h after NP addition (Figure 3B). Effects on *Il12b* expression were moderate; however, there was a significant increase after 24 h in macrophages treated with 100 µg/mL 50 nm NPs (Figure 3C).

Costimulatory molecules are important mediators for immune cell activation upon encounter with foreign particles. When evaluating the mRNA expression of costimulatory molecules in macrophages that received 25 nm NP particles, we observed a marked decrease in *Cd80* mRNA levels after 2 h (Figure 3D). Notably, this effect gradually normalized with longer incubation (Figure 3D). Similar effects were observed with 50 nm particles, although the recovery time was not as evident (Figure 3D). Interestingly, despite the early suppressive effect of NPs on *Cd80* mRNA expression, *Cd80* expression significantly increased after 24 h incubation with 50 nm particles (Figure 3D). *Cd86* levels followed a somewhat opposite pattern to that observed for *Cd80*. *Cd86* expression increased after incubation with 25 nm and 50 nm NP particles for 2 and 4 h and then gradually declined (Figure 3E). Interestingly, *Cd86* expression levels significantly decreased after 24 h incubation with the lowest and highest particle dose, which might indicate macrophage exhaustion (Figure 3E).

### 3.3. Long-Term NP Administration Does Not Affect the Gastrointestinal Homeostasis

Given these clear effects on macrophages in vitro, we investigated whether NP administration impacts gastrointestinal health and homeostasis. Freshly-weaned WT mice were exposed to 25 nm PMMA particles in their drinking water at a concentration of 0.05 mg/mL for 6 months.

**Figure 4:**
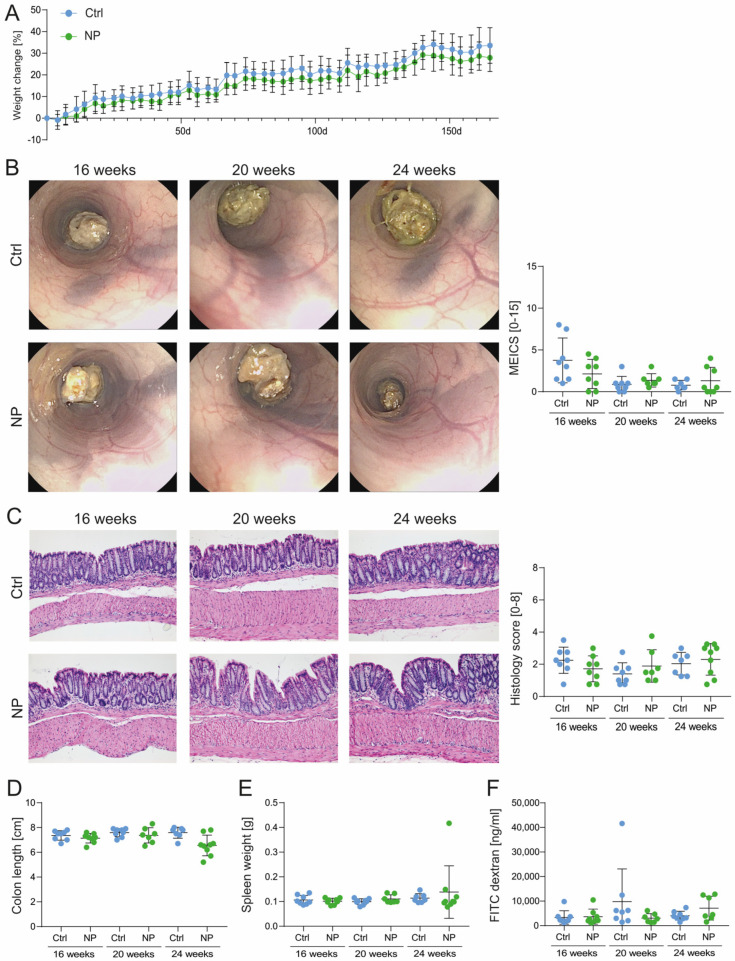
NPs did not affect intestinal homeostasis. Directly after weaning, female WT mice were supplemented with 25 nm PMMA particles in the drinking water (0.05 mg/mL) for 172 days (6 months). (**A**) Weight development during pretreatment. (**B**) Representative colonoscopy images and MEICS scores. (**C**) H&E staining of distal colon sections with analysis of epithelial damage and infiltration. (**D**) Colon length and (**E**) spleen weight. (**F**) Epithelial barrier integrity assessed by quantifying FITC-dextran levels in serum 5 h after oral administration of 4 kDa FITC-dextran. (**A**,**B**) Two-way ANOVA with Tukey’s multiple comparisons. (**B**,**C**) Kruskal–Wallis test. (**D**–**F**) One-way ANOVA. Original magnification (H&E) ×10.

Long-term NP administration did not affect weight development (Figure 4A). Colonoscopy (Figure 4B) and histology (Figure 4C) did not show intestinal abnormalities that would indicate low-grade inflammation of the colon. Additionally, there were no differences observed in colon length (Figure 4D) or spleen weight (Figure 4E), and barrier integrity was not altered in NP-treated mice compared to the controls (Figure 4F). These findings indicate that the ingestion of 25 nm PMMA particles does not alter gut homeostasis or provoke spontaneous intestinal inflammation in healthy WT mice.

### 3.4. Long-Term NP Administration Does Not Aggravate DSS-Induced Colitis

Having shown that NP administration does not have adverse effects on gastrointestinal health during homeostasis, we aimed to investigate whether the presence of NP particles in the diet might impact the development of intestinal inflammation. For this aim, freshly weaned WT mice were treated with 25 nm PMMA particles for 6 months before induction of acute DSS colitis.

**Figure 5:**
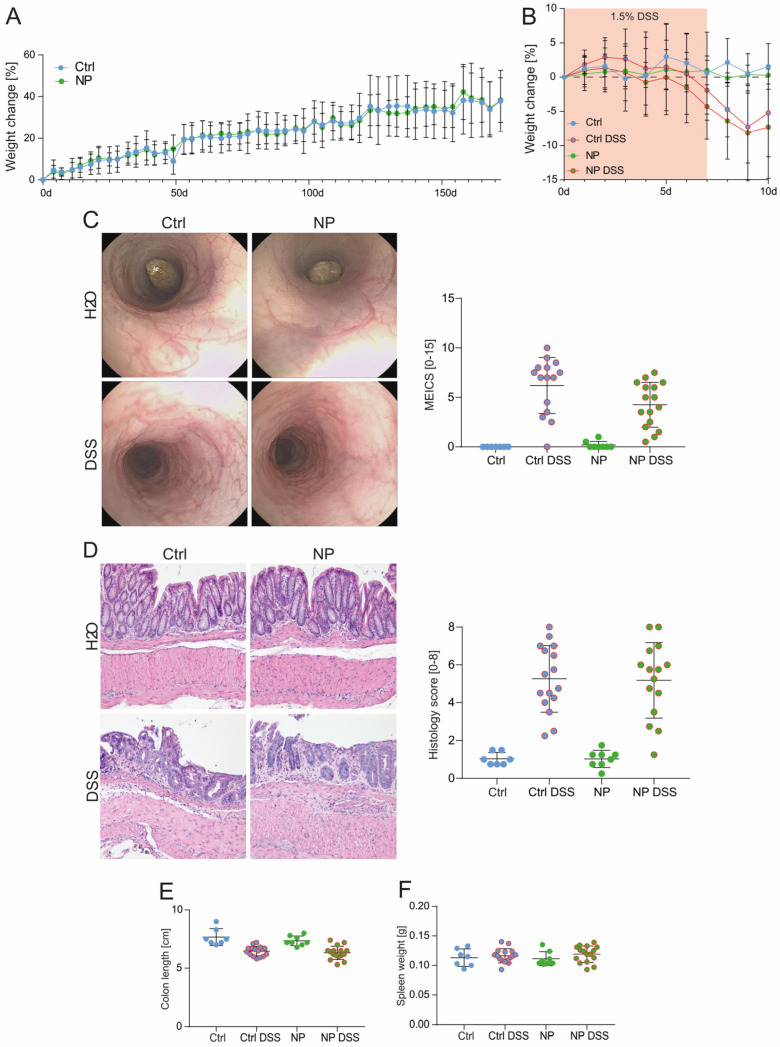
Administration of NP particles did not exacerbate acute DSS colitis. Directly after weaning, female WT mice were supplemented with 25 nm PMMA particles in the drinking water (0.05 mg/mL) for 172 days (6 months). Acute colitis was induced by administration of 1.5% DSS in the drinking water for 7 days. (**A**) Weight development during NP pretreatment and (**B**) colitis induction. (**C**) Representative images from colonoscopy and MEICS score. (**D**) H&E staining of distal colon sections and analysis of epithelial damage and infiltration. (**E**) Colon length and (**F**) spleen weight. (**A**,**B**) Two-way ANOVA Tukey’s multiple comparison (**C**,**D**) Kruskal–Wallis Test. (**E**,**F**) One-way ANOVA. Original magnification (H&E) ×10.

Throughout the entire experiment, 25 nm nanoparticle particles were continuously added to the drinking water at a concentration of 0.05 mg/mL. Consistent with our findings from long-term exposure, NP administration did not affect weight gain (Figure 5A). Following the administration of DSS for seven days, weight loss was comparable across all groups regardless of NP administration (Figure 5B). Neither colonoscopy (Figure 5C) nor histology analysis (Figure 5D) revealed aggravation of DSS-induced colitis upon NP administration. No differences in colon length (Figure 5E) and spleen weight (Figure 5F) were observed among the individual treatment groups. Thus, we conclude that NP ingestion impacts neither intestinal homeostasis nor the development of intestinal inflammation. Using a previously established protocol [35], Raman spectroscopy was incapable of detecting the administered PMMA NP particles in the intestine and distant organs (Figure S2). There were no significant differences observed when comparing the Raman spectra of tissue digests from NP-treated groups to their respective controls.

## 4. Discussion

Plastic pollution poses one of the biggest challenges of our time. Despite growing evidence that the plastic contamination in our food might be more pervasive than expected [12,13,14,15,16,17,18], there have been limited studies exploring the effects of ingested plastic particles on human health, and data on NP particles are very controversial. With plastic pollution escalating worldwide, it is evident that understanding the effects of plastic exposure on the human body, particularly on the digestive system, is crucial. In this study, we examined the influence of NP particles on gastrointestinal health both in vitro and in vivo.

Our study investigated the impact of nanoparticle (NP) exposure on bone-marrow-derived macrophages (BMDMs) and its implications for gastrointestinal health. The exposure of BMDMs to 25 nm PMMA or 50 nm PS particles led to notable morphological changes, including a shift to a spherical shape and increased detachment from culture plates, suggesting that NP particles can substantially modify the cellular structure of macrophages. In addition to morphological changes, NP exposure also triggered an inflammatory response in BMDMs. Contrary to the in vitro findings, long-term exposure to 25 nm PMMA particles in drinking water did not adversely affect gastrointestinal health or homeostasis in WT mice. Measures of weight gain, colonoscopy, histology, and epithelial barrier integrity remained unchanged compared to controls. Furthermore, NP administration did not exacerbate acute colitis induced by DSS, with similar outcomes in weight loss, colon length, and spleen weight across treatment groups. These results suggest that while NP exposure induces significant inflammatory and morphological changes in BMDMs in vitro, it does not appear to disrupt gastrointestinal homeostasis or worsen DSS-induced colitis in vivo. This discrepancy might be attributed to the differences in NP concentrations, exposure duration, or the complexity of the in vivo environment compared to cell culture conditions. Additionally, Raman spectroscopy did not detect NP particles in the intestine or distant organs, indicating that the particles might not persist or accumulate in significant amounts. Overall, our findings provide insight into the cellular impact of NP exposure and suggest that while NP particles can induce an inflammatory response in macrophages, their long-term ingestion does not necessarily impair gastrointestinal health or exacerbate inflammatory conditions in mice. Further studies are warranted to explore the broader implications of NP exposure on different biological systems and chronic health outcomes.

Macrophages respond to foreign particles that breach the intestinal epithelial barrier by engulfing and clearing them from the system through phagocytosis [25]. Here, we showed that NPs engulfed by macrophages cause morphological alterations in these cells. Moreover, NP administration leads to increased expression of proinflammatory cytokines such as *Tnfa*, *Il6*, and *Il12b*, along with alterations in the expression profiles of costimulatory molecules *Cd80* and *Cd86*. These changes indicate a proinflammatory response following exposure to plastics. In line with our experiments, a recent study by Banerjee et al. reported cell death due to oxidative stress, membrane damage, immune response, and DNA damage in mammalian cells treated with NP or MP particles [36]. Those adverse effects increased significantly with small particle size, the positive charge of the particles, high administration dose, and longer incubation times and were more severe when the particles were loaded with surfactants or adsorbed pollutants [36]. The process of engulfing nanoplastics might alter the physical state of macrophages, affecting their cytoskeleton and, thus, their ability to migrate. The energy and resources spent on phagocytosing nanoplastics might also reduce their capacity to move and respond to other stimuli. This might have severe impacts on macrophage function and innate immune response.

Contrary to the strong effects on macrophages observed in vitro, long-term NP administration in vivo had no adverse impacts on the gastrointestinal health of mice, nor did it aggravate experimental intestinal inflammation. Previously, plastic research predominantly centered on the impacts of plastic contamination in marine and freshwater ecosystems, using fish, mollusks, or crustaceans as model organisms [37], whereas information on the toxicity of NP particles in mammalian systems is highly limited. Initial studies investigating the effects of NP ingestion in mice reported alterations of gut microbiota and mucus secretion [27,38], impairment of the barrier integrity [39,40], intestinal damage, acute inflammation, and oxidative stress [41,42,43]. Yang et al. recently reported aberrant macrophage polarization and activation upon PS nanoplastic exposure, resulting in IL1β secretion, altered T-cell response, and, ultimately, adverse impacts on brain function [44,45]. However, these studies used relatively high amounts of plastic particles within a short duration, which may not reflect realistic conditions. In contrast, we opted for physiological conditions that closely mimic the in vivo situation observed in healthy individuals or patients suffering from inflammatory bowel disease (IBD). In our studies, we used methacrylate particles, which find use as raw material in the production of many plastics. In humans, exposure to plastic typically begins within the first few months after birth, as babies are fed milk and, later, other beverages from plastic baby bottles [46]. The first and most significant peak in the onset of IBD occurs in late adolescence and early adulthood at the age between 15 and 30 years. Building on these observations, we initiated pretreatment of mice with NP and MP particles immediately after weaning and induced acute DSS colitis following long-term exposure to plastic particles for 6 months. The presence of MPs in placental tissue suggests that plastic exposure may even commence during fetal development through the transfer of small plastic particles from mother to unborn child [47], raising significant concerns. Our study design was based on the infamous data extraction study conducted at the University of Newcastle, Australia, which indicated that individuals later consume approximately 5 g of small plastic particles per week, totaling over 250 g annually [18]. Scaling down this amount to the average size of a mouse, we administered a corresponding daily dose of 0.2 mg of NP particles. For our model, we chose to administer plastic particles via drinking water to enable consistent distribution of the dose throughout the day. However, estimates of human plastic consumption vary widely depending on the methodologies used in different studies. While some research suggests that humans consume small but significant amounts of plastic weekly, others argue that these estimates may be imprecise or based on limited data. Furthermore, it is not known which fraction of the ingested plastic is nanosized. Moreover, unlike our model, real-life human exposure involves a dietary-related mixture of NP and MP particles of varying sizes and types, although there is currently no data available on the composition of the ingested plastic burden. Nevertheless, it would be interesting to test how a mixture of different plastic particles coated with other materials such as pollutants or heavy metals might affect intestinal and whole-body health.

Detection of micro- and, especially, nanosized plastic particles in human or murine tissue remains challenging, and plastic research is lacking uniform and consistent methods. Despite intense research on the development of plastic particle detection and identification methods, there is currently no reliable and well-established methodology available that would enable the detection of PMMA nanoparticles in murine tissue. Therefore, we cannot conclude whether the absence of in vivo effects is a result of the possibility that the NP particles are not surpassing the intestinal epithelium and are subsequently excreted, or whether they are penetrating into the mucosa and simply do not affect gut homeostasis or inflammation—this is certainly the biggest limitation of this study. While Raman spectroscopy enabled the detection of NP and MP particles in tissue lysates in a previous study from our lab [35], the administered PMMA particles could not be evidenced clearly following the same approach. This might be attributed by the chemical structure differences between PMMA and PS. PS contains a phenol ring, which results in a prominent Raman peak at approximately 1000 nm Raman shift, while PMMA does not exhibit such a characteristic intense peak. With the development of advanced spectral analysis techniques, such as machine learning-based algorithms, it may be possible in the near future to accurately identify these polymers even in cases where their characteristic peaks are not as pronounced.

## 5. Conclusions

Plastic pollution is a significant environmental issue, and evidence suggests that plastic contamination in food is widespread. Our study examined the effects of nanoparticle (NP) exposure on gastrointestinal health. In vitro, 25 nm PMMA and 50 nm PS particles caused notable changes in macrophage morphology and inflammation. However, long-term exposure to 25 nm PMMA in mice did not impact gastrointestinal health or worsen colitis. Hence, the fact that NP ingestion did not affect the severity of experimental colitis in our animal model can be considered a relief for patients suffering from inflammatory bowel diseases. Nevertheless, individual lifestyle choices and dietary preferences may contribute to increased plastic ingestion. Therefore, considering the potentially facilitated uptake of environmental toxins, long-term effects on the organism and gastrointestinal health cannot be excluded.

## Data Availability

The original contributions presented in the study are included in the article/supplementary material; further inquiries can be directed to the corresponding author.

## Supplementary Materials

The following supporting information can be downloaded at: https://www.mdpi.com/article/10.3390/nano14161350/s1, Figure S1: Characterization of PS particles used for in vivo experiments; Figure S2: Raman spectroscopy.
