# Supplementary material for: Impact of Nanoplastic Particles on Macrophage Inflammation and Intestinal Health in a Mouse Model of Inflammatory Bowel Disease
Source: Nanomaterials (Basel). 2024 Aug 15;14(16):1350. doi: 10.3390/nano14161350 (PMC11357591; doi:10.3390/nano14161350)
Supplement: Supplementary file 1 [file nanomaterials-14-01350-s001.zip › nanomaterials-3120680-supplementary.pdf]

SUPPLEMENTARY MATERIAL AND METHODS

1. Transmission electron microscopy

For Transmission electron microscopy (TEM) analysis, PS suspensions were either diluted (1:200) and a drop of the diluted suspension was drawn through a poly-L-lysine (PLL) functionalized lacy carbon TEM grid (1 µm PS particles). The 50nm PS particles were diluted 1:200 and centrifuged on (PLL) functionalized TEM grids (1 hour, 14,000 g). The particles were imaged using a scanning transmission electron microscope (HD2700Cs, Hitachi, Japan) operated at an acceleration voltage of 200 kV. For image formation, either a secondary electron (SE) or a high angular annular dark field (HAADF) detector was used.

2. Dynamic light scattering

The hydrodynamic diameter of the 25 nm PMMA particles was determined using dynamic light scattering (DLS, nanoZS, Malvern Panalytical, United Kingdom). For that purpose, stock suspensions were diluted in the drinking water that was offered to the animals at a concentration of 0.05 mg/ml. The size of the particles was measured daily over the course of 48 h. After 24h the suspension was manually turned upside down a few times. Selected measurements were also conducted before and after turning the samples upside down. Measurements were conducted after 0 h, 24 h, and 48 h.

3. Tissue digestion

Organs were digested according to Thiele *et al.* [1] using 40% KOH (3 x tissue volume; Sigma-Aldrich, Missouri, United States) for 48 h at 40°C (no agitation). Digestion was stopped with 1 M citric acid (Sigma-Aldrich), digests were filtered through 5 µm PVDF membranes (Merck, Darmstadt, Germany), and one drop of the filtrate was put onto a glass slide. Polystyrene contamination was avoided at any time. Once the liquid was evaporated, the filtrate was investigated using Raman Spectroscopy.

4. Raman spectroscopy

Raman spectroscopy was performed in Horiba LabRAM HR Evolution with the laser (785 nm; source power: 300 mW), grating (grooves density: 300 l/mm), objective lens (Olympus MPlanN, NA=0.9), and detector (Horiba Synapse CCD). The grating was calibrated by reference Silicon peak using the laser, and optical filters (edge filter and holographic notch filter) were placed in the Raman beam path to selectively block the laser line (Rayleigh scattering) while allowing the Raman scattered light through to the detector. The spectra were acquired in Raman shift range 350-1800 cm^{-1} with three times accumulation for averaging spectra and filtering spike. We ensured the absence of sample damage by visual inspection after imposing the laser. Pure PMMA NP (Fig. S2A) and digestion buffers ± filtering (Fig. S2B,C) were included as controls. Raman Spectroscopy was performed on tissue samples of three randomly chosen mice per condition (n=3).

Supplementary Figures

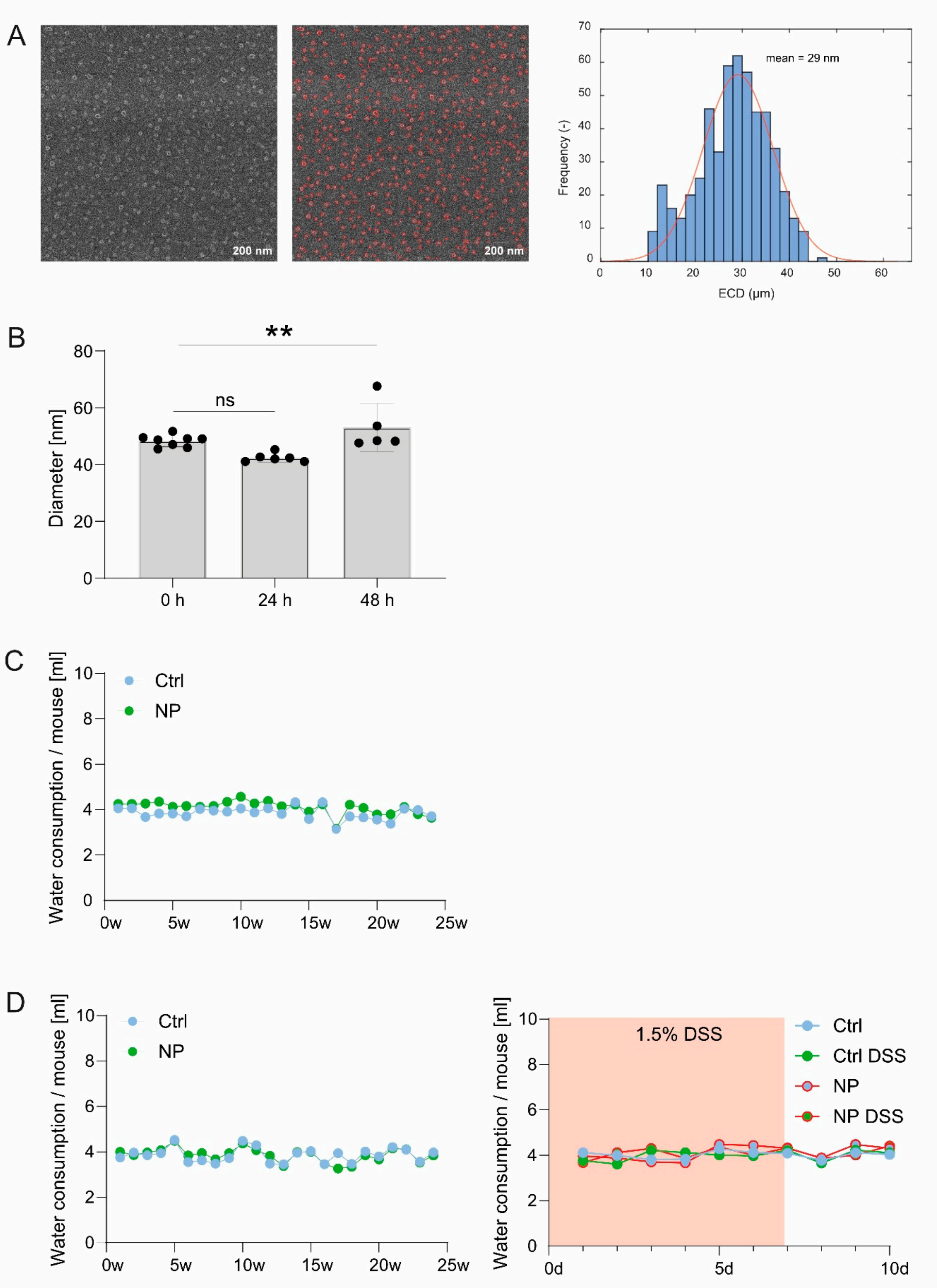

Figure S1. Characterization of PS particles used for *in vivo* experiments. A) TEM images of 25 nm PMMA particles taken with a secondary electron (SEM) or a high angular dark field (HAADF) detector after 48 h. B) PS particles were diluted according to our *in vivo* setup (0.05 mg/ml in drinking water from the animal facility) and the suspension was analyzed using dynamic light scattering. Measurements were conducted after 0 h, 24 h, and 48 h. One way ANOVA with Tukey's multiple comparison. Water consumption per mouse during NP administration

corresponding to experiment shown in C) Figure 4 and D) Figure 5.

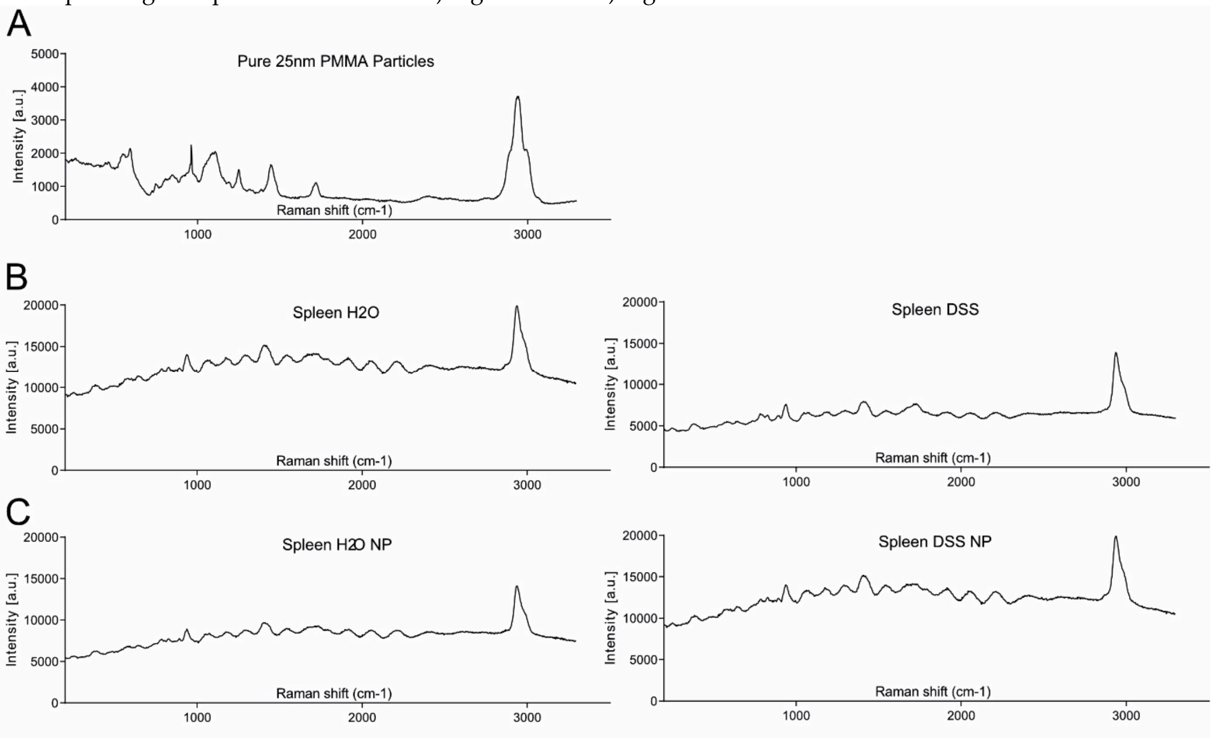

Figure S2. Raman Spectroscopy. Right after weaning, female WT mice were supplemented with 25 nm PMMA particles in the drinking water (0.05 mg/ml, daily intake: 0.2 mg at average water consumption of 4 ml/day) for 166 days (>6 months). Organs were digested in KOH and tissue digest was analyzed for accumulation of plastic particles using Raman spectroscopy. A) Raman Spectra of pure 25nm PMMA NP particles supplemented during in vivo experiments. B) Raman Spectra of spleen digests from H2O Ctrls and NP-treated mice. C) Raman Spectra of spleen digests from DSS Ctrls and DSS NP-treated mice. Tissue samples of three randomly chosen mice were analyzed per condition (n=3).
